# Youth development under digital surveillance: challenges, opportunities, and future directions

**DOI:** 10.3389/fsoc.2026.1811778

**Published:** 2026-05-08

**Authors:** Artur Ishkhanyan, Mane Hakobyan

**Affiliations:** 1 Presidium, National Academy of Sciences of the Republic of Armenia, Yerevan, Armenia; 2 Institute for Physical Research, Ashtarak, Armenia

**Keywords:** digital literacy, digital surveillance, emotional regulation, identity formation, platform governance, youth development

## Abstract

Digital technologies now operate as developmental infrastructures, shaping how young people learn, relate, and form identities. This paper develops a Vygotskian framework for analyzing youth surveillance, complemented by Foucauldian and Deleuzian perspectives on power and control. We argue that contemporary surveillance systems do not simply observe youth but reorganize the relational and cultural conditions of development. Drawing on research across three analytical layers—situated environments, systemic infrastructures, and transformative horizons—we show how surveillance channels developmental pathways toward legibility and prediction. At the same time, youth enact situated forms of agency that reconfigure these systems. The paper concludes with policy and design implications that foreground developmental dignity, epistemic justice, and youth co-authorship of digital environments.

## 1 Introduction

The digital age has fundamentally reconfigured the architecture of youth development, transforming technologies of connection into infrastructures of governance that permeate the most intimate spheres of growth (Zuboff, 2019). What began as tools for creativity and social engagement now operate as pervasive surveillance systems, shaping behaviors, relationships, and identities from early childhood through adolescence (Livingstone and Blum-Ross, 2020). This shift extends beyond data collection to what this paper terms the algorithmic colonization of developmental pathways: the redirection of human growth into quantifiable, trackable, and optimizable data streams under digital capitalism (Andrejevic, 2013; Beer, 2017). The result is a reconfiguration of the historical conditions of developmental mediation. Where once behavioral oversight was bounded by institutional spaces such as home and school, today's ambient surveillance dissolves these boundaries, producing fluid regimes of observation and modulation (Deleuze, 1992). This paper advances a central claim: digital surveillance should be understood not merely as observation, but as a form of developmental governance that reorganizes the relational, cultural, and institutional conditions of human development.

In this paper, surveillance refers to the systematic collection and interpretation of behavioral, biometric, or interactional data for governance purposes. Digital surveillance denotes technologically mediated forms of such practices, while surveillance technologies refer to the socio-technical systems—platforms, sensors, and algorithms—through which these processes are enacted. These definitions clarify the conceptual scope within which the theoretical framework of the paper is articulated.

This argument is developed through a developmental framework centered on Lev Vygotsky's theory of social mediation, which conceptualizes development as a relational process emerging through guided participation and shared cultural practices (Vygotsky, 1978). From this perspective, surveillance does not merely observe behavior—it reshapes the scaffolding through which development unfolds. Michel Foucault's notion of biopower helps illuminate how surveillance technologies normalize certain developmental trajectories while rendering others deviant (Foucault, 1977, 1978). Meanwhile, Gilles Deleuze's concept of control societies highlights the shift from spatial discipline to continuous modulation through data flows and behavioral nudges (Deleuze, 1992). Together, these perspectives clarify how digital infrastructures reconstitute the developmental environment itself. While surveillance systems exert structuring pressures, their effects remain contingent on institutional contexts, cultural norms, and youth agency. This paper therefore avoids technological determinism and instead conceptualizes surveillance as a contingent and contested form of governance.

Surveillance is unevenly distributed across social and geopolitical contexts. Biometric systems are often deployed most intensively among marginalized youth, reflecting asymmetries in state priorities, cultural valuations of privacy, and corporate influence (Eubanks, 2018; Taylor and Rooney, 2016). Although the technical architectures are novel, the politicization of youth development is not. As a historical contrast, the Soviet youth project provides a civic, collectivist model of developmental governance; it is used throughout the paper as a counterpoint that highlights how different developmental systems—past and present—structure identity formation, responsibility, and belonging (Gorsuch, 2000). This comparison is not intended to idealize Soviet systems, but to contrast different modes of developmental mediation. Unlike contemporary algorithmic infrastructures, which rely on statistical inference and opacity, earlier systems used narrative, ritual, and relational pedagogy. Juxtaposing these models helps distinguish what is genuinely new about today's algorithmic childhood.

This study adopts a conceptual-synthetic methodology. It draws on interdisciplinary literature in sociology, education, and surveillance studies, using illustrative examples from existing research rather than original empirical data.

*These examples are drawn from academic scholarship, official documents, policy reports, NGO publications, and credible news media, and are used to demonstrate analytically relevant recurring patterns rather than to provide representative statistical evidence*.

The analytical domains examined across the paper's three layers were identified through iterative thematic synthesis, combining engagement with key theoretical frameworks (Vygotsky, Foucault, Deleuze) and inductive clustering of recurring patterns in the literature.

To analyze these dynamics, the paper examines seven interrelated domains through which developmental governance is being reshaped: domestic life, youth resistance practices, educational environments, digital infrastructures, cultural epistemologies, governance and policy frameworks, and emerging neurotechnologies. These domains are not empirical categories but analytical lenses that capture distinct sites in which developmental governance is enacted. Taken together, they are organized into three analytical layers: situated environments (domestic and educational life), systemic infrastructures (digital ecosystems and governance), and transformative horizons (cultural perspectives, resistance, and emerging neurotechnologies).

Clarifying development as a theoretical anchor is essential. In this paper, development refers to the relational, contextual, and culturally mediated processes through which children and adolescents acquire capacities, identities, and forms of participation (James and Prout, 2015). Digital surveillance reshapes these processes by altering the conditions of social mediation: who guides learning, what kinds of behaviors are recognized or penalized, and how young people interpret themselves in relation to others. As Vygotsky noted, development is always co-authored; under surveillance, that co-authorship is increasingly shared with algorithmic systems, predictive classifiers, and complex data-driven environments.

Theoretically, this framework advances the concept of developmental governance—a lens that reframes surveillance not as data extraction but as a structuring force that reorganizes the relational, institutional, and cultural conditions of youth development. For digital sociology, this shifts attention from platform architectures to their developmental mediation; for sociology of education, it reframes algorithmic systems not as instructional tools, but as actors that reshape the very possibility of pedagogy.

The aim of the paper is therefore twofold: first, to demonstrate how surveillance technologies reorganize the developmental ecologies in which young people grow; and second, to advance a framework that recognizes youth as meaning-makers who negotiate, reinterpret, and resist these systems. The analysis highlights the need for developmental infrastructures that protect relational autonomy and honor diverse epistemologies. Ultimately, the paper advocates for a reimagined social contract—one in which youth participate as co-authors of the ethical terms under which digital systems shape their becoming.

*To develop this argument, the paper proceeds across three analytical domains. It first examines situated environments in which surveillance enters everyday developmental life, particularly the home and school. It then turns to systemic infrastructures through which governance is institutionalized across digital ecosystems and policy regimes. Finally, it explores transformative horizons in which cultural difference, youth resistance, and emerging neurotechnologies reshape the future conditions of development*.

## 2 Situated environments of developmental surveillance

### 2.1 Domestic surveillance

The transformation of domestic life into a terrain of continuous monitoring marks a shift in childhood development. Once a site of relative sanctuary, the home has become an extension of institutional oversight, embedding surveillance into everyday routines. Behaviorist legacies endure in new forms: John B. Watson's suspicion of maternal affection and B.F. Skinner's token economies now animate smart bassinets and platforms like ClassDojo, where compliance is tracked, scored, and shared in real time (Watson, 1928; Williamson, 2017). Smart toys record speech, GPS trackers monitor movement, and educational apps harvest biometric data—all normalized under the rhetoric of innovation and care (Livingstone and Smith, 2014). The pandemic further dissolved the spatial boundaries between home and school, accelerating the incorporation of platforms like Google Classroom into domestic routines (Lupton and Williamson, 2017). For children and adolescents, development increasingly unfolds within these surveilled ecologies, where everyday interactions are rendered into data.

These developments can produce what this paper terms *visibility coercion*: the unspoken condition whereby access to education, safety, or basic services increasingly requires data surrender—a dynamic that disproportionately burdens those with fewest alternatives. In India, government-issued tablets come pre-installed with keystroke-tracking software, while in the United States school-provided Chromebooks equipped with GoGuardian monitor student activity in real time. In Kenya, biometric attendance systems determine eligibility for subsidized meals. Disabled adolescents report being tracked by welfare agencies as a condition for support, reducing biological existence to a metric of eligibility (Eubanks, 2018; Agamben, 1998). These cases can be read as resembling a digital variant of the colonial gift economy: technological inclusion predicated on surveillance compliance (Philip et al., 2011).

Cultural norms shape how these systems are adopted and contested. U.S. parents often embrace monitoring tools as expressions of individual responsibility and risk aversion (Boyd, 2014; Livingstone and Blum-Ross, 2020). In contrast, Japanese parents emphasize anshin (emotional assurance) over panoptic certainty, favoring symbolic check-ins over live tracking (Livingstone and Third, 2017). Scandinavian models push back against surveillance through a blend of infrastructural transparency and high social trust (Boyd, 2014). These contrasts underscore that surveillance technologies are not culturally neutral—they are interpreted through value systems that shape their legitimacy and limits, and thus the developmental meanings young people attach to being watched.

Surveillance in the home is not unilaterally imposed. It is often co-constructed and mediated by intergenerational actors. Parents may act as algorithmic enforcers—monitoring app scores, imposing screen-time penalties—or as quiet resisters who disable tracking features or challenge school mandates. In multigenerational households, older siblings or grandparents may serve as interpreters of digital risk, offering guidance grounded in context and care. This mediation underscores that developmental surveillance is negotiated through relational ecologies—not simply imposed from above, but continually filtered, normalized, and contested through everyday intersubjective dynamics.

Within these architectures, youth engage in *algorithmic comportment*: the modulation of emotional and physical expression to comply with machine-readable norms, often internalizing the logic of surveillance as a condition of participation. Some suppress signs of anxiety to avoid triggering alerts; others adjust their gait or mask stimming behaviors to avoid biometric detection (Hope, 2016). These adaptations impose psychological costs, privileging performance over authenticity and embedding self-monitoring into the texture of everyday development (Shade and Singh, 2016).

Yet youth also resist. Across diverse contexts, young people are innovating strategies of subversion—from spoofing biometric systems to encrypting communications and repurposing school devices for unsanctioned uses—often structured by material inequities and differential access to technical literacies (Selwyn, 2021; Shade and Singh, 2016; Taylor and Rooney, 2016). These practices are explored in greater detail in Section 4.2, which examines how such tactics become part of the social learning through which youth acquire what might be called resistance literacies.

Play, once a protected domain of unstructured exploration, has also been absorbed into surveillance logics. Apps like Zamzee transform movement into data; recess is gamified through Fitbit-style mandates; and platforms reward aesthetic compliance over expressive experimentation (Benjamin, 2019; Gray, 2011; Marsh et al., 2016). Youth curate multiple digital selves: a public, biometric self visible to school platforms and a more private self maintained elsewhere. For neurodivergent teens, in particular, this toggling between visibility and concealment can induce identity fragmentation and cognitive fatigue, as documented in qualitative studies of masking and self-monitoring in neurodivergent communities (Neurodivergent Insights, 2022).

Parents, especially mothers, experience surveillance as emotional labor. In lower-income communities, state-distributed technologies often eliminate refusal as a viable option, coercing caregivers into complicity with tools they distrust (Berriman and Thomson, 2015; Eubanks, 2018). These dynamics raise pressing questions about structural interventions. Rather than detailing policy mechanisms here, Section 3.2 elaborates solutions such as encryption redistribution, algorithmic audit rights, and youth-governed privacy standards that can re-balance power within domestic and educational infrastructures.

Above all, preserving ambiguity and dissent within domestic life is essential for development. As Peter Gray (2011) argues, unsupervised play fosters resilience and creativity. Without space for opacity, the algorithmic redefinition of home risks foreclosing the very conditions that make youth development human: experimentation without constant evaluation, relational trust without permanent capture, and the possibility of becoming otherwise than what the data predicts.

*These domestic dynamics do not end at the household threshold; they extend into educational institutions, where similar surveillance logics increasingly shape learning and authority*.

### 2.2 Educational praxis and algorithmic learning

The classroom, once governed by interpersonal interaction and recognizable pedagogical norms, is increasingly organized through opaque infrastructures of algorithmic evaluation. Platforms such as ClassDojo, GoGuardian, ExamSoft, and Google Classroom embed engagement metrics and predictive scoring into everyday routines. These systems shift the criteria for what counts as learning, privileging measurable behaviors and visible activity over interpretive depth, collaborative deliberation, and exploratory inquiry (Monahan and Torres, 2010; Roberts-Holmes and Bradbury, 2016).

The developmental implications are substantial. In some documented “smart school” implementations in China, posture-analysis cameras can automatically deduct points for perceived inattentiveness (Kostka, 2019). In contexts such as Brazil, where facial-recognition systems have been deployed in educational or public infrastructures, concerns have been raised about disproportionate impacts on Black and Indigenous youth, reflecting broader patterns of racial bias in computational systems (Benjamin, 2019). Rather than simply recording behavior, such tools script normative pathways for students, shaping how attentiveness, discipline, or academic promise are defined.

In Vygotskian terms, development unfolds through shared relational space and dialogic exchange. Automated cues redirect that space toward compliance with platform logic. Students increasingly respond not to teachers' interpretive guidance but to the thresholds embedded in algorithms. Inquiry gives way to the imperative of remaining machine-readable.

These dynamics can be especially difficult for neurodivergent learners. Adolescents with ADHD or autism describe the strain of adapting their bodily rhythms—eye contact, motion, stimming—to match the behavioral parameters inferred by automated tools as documented in qualitative accounts of masking and self-monitoring among neurodivergent youth. In contexts such as India, scholars have raised concerns that students from marginalized caste backgrounds, including Dalit communities, may be disproportionately flagged as disengaged when their culturally situated communication styles diverge from platform expectations. In Canada, disability advocates have documented how behavioral analytics embedded in individualized learning plans penalize students for natural fluctuations in attention or movement (Coghlan et al., 2020). In such cases, difference is often rendered as misclassification: platform infrastructures recode neurodiversity and cultural variation as deviations from a computational norm.

A historical comparison clarifies what is distinctive about these systems. Even highly ideological educational regimes—such as those of the Soviet Pioneer and Komsomol organizations—relied on interpersonal mentorship, moral narratives, and collective ritual rather than automated abstraction. The point is not to valorize these earlier models but to note that developmental formation was mediated through human interpretation and relational practices. While historically distinct, this comparison highlights differences in mediation rather than normative superiority. Contemporary systems, by contrast, outsource interpretation to automated scoring routines that flatten nuance and convert ambiguity into risk or noncompliance.

Another emerging trend is the rise of algorithmic therapeutic governance: emotion-sensing AI that interprets facial micro-expressions or physiological signals as indicators of stress, disengagement, or disorder. Marketed as tools for care, such systems frequently misinterpret culturally distinct affect, neurodivergent expression, or the ordinary fluctuations of adolescence. As Foucault (1977) cautioned, when diagnostic functions merge with disciplinary power, intervention easily masquerades as protection. In such environments, emotional life becomes subject to automated interpretation, narrowing the space in which students can experiment with identity, affect, and self-understanding.

Teachers, too, are reshaped by these infrastructures. According to documented reports, rural students in Colombia have been flagged as cheating due to internet instability, prompting teachers to manually adjust data to protect them from punitive flags. Such practices reveal how pedagogical relationships become entangled with the need to satisfy platform metrics. As educators redirect attention from dialogic learning to maintaining compliance with automated systems, the relational foundations of teaching—trust, responsiveness, and shared interpretation—are strained.

Nevertheless, counter-practices offer alternative models. In contexts such as South Africa, global platforms like LabXchange have been adapted to incorporate local languages such as isiZulu, anchoring digital materials in cultural and linguistic specificity. Colombian teachers have developed “shadow rubrics” that quietly supplement formal scoring with criteria emphasizing creativity and ethical deliberation, intentionally shielded from automated assessment. Barcelona's Decidim initiative invites students into the co-design of civic technologies, enabling them to participate in defining how digital tools shape learning (Calzada, 2021). These examples reclaim the classroom as a space of collective negotiation rather than automated evaluation.

Emerging reforms in jurisdictions such as Norway, Ontario, and Chile suggest that statutory protections and participatory evaluation procedures can help counterbalance automated platforms and restore conditions for intellectual autonomy. These interventions underscore a central point: development is more than the accumulation of measurable behaviors—it is a culturally situated, relational process that cannot be reduced to data extraction or predictive classification.

Ultimately, educational praxis must reaffirm its ethical commitments. Resistance literacy—the ability to recognize, interpret, and critique the governance encoded in digital systems—is not an accessory to digital competence but its essential foundation. Students must be taught to understand how infrastructures shape possibility, how data traces can constrain interpretation, and how systems can be redesigned to support, rather than delimit, their developmental trajectories. As Freire (1970) insisted, freedom in education arises not from compliance but from critical consciousness: the capacity to question, reinterpret, and imagine different futures. Operationalizing this commitment requires limiting automated behavioral scoring, reinforcing interpretive teacher authority, and integrating critical data literacy into curricula.

*These situated environments do not operate in isolation. They are structured by wider technical architectures, market arrangements, and regulatory frameworks that determine how surveillance systems are deployed, normalized, and contested*.

## 3 Systemic infrastructures of developmental governance

### 3.1 Digital ecosystems and developmental infrastructure

Whereas the preceding section focused on classroom practices, this section examines the infrastructural architectures that shape developmental possibilities at a systemic level. Digital surveillance infrastructures are not passive technical supports but formative environments that shape how young people learn, participate, and imagine their futures. They embed particular assumptions about control, value, and social order, thereby configuring the institutional terrain in which development unfolds (Ruckenstein and Schüll, 2017). Echoing earlier biometric traditions, contemporary educational platforms render students interpretable to algorithms through standardized metrics, reorganizing the conditions under which learning is perceived and legitimized. The figure of the “datafied child” (Lupton and Williamson, 2017) is emblematic of this shift: a learner whose capacities are inferred through data-driven analytics rather than cultivated through situated, relational engagement.

National digital strategies often amplify these logics. In contexts such as India, policy frameworks including the National Education Policy 2020 have been analyzed as promoting adaptive learning systems and nationwide digital benchmarks that can obscure the richness of localized knowledge traditions (Selwyn, 2021; Williamson, 2017). Kenya's student biometric systems have been examined as connecting identity verification with access to services, embedding a model of surveillance-for-access into public education (Weitzberg, 2020). In Mexico, scholars have raised concerns about public–private partnerships that package connectivity with proprietary software, introducing opaque data practices under the guise of digital inclusion. Across such contexts, infrastructural design produces new conditionalities: access to learning or welfare is increasingly mediated by one's algorithmic visibility.

As Deleuze (1992) suggested, contemporary systems operate less through explicit discipline and more through subtle modulation—recalibrating behavior by structuring the informational environment itself (Beer, 2017; Cook et al., 2009). These dynamics have developmental consequences: the broader ecologies in which children gain competencies and identities are conditioned by what infrastructures permit, reward, or penalize. Whereas the preceding discussion of educational praxis addressed the immediate pedagogical interface, this section focuses on the deeper systemic architectures that define what forms of communication, relationality, and cultural expression are institutionally enabled.

These infrastructures encode normative assumptions into software logic, procurement requirements, and data architectures. Design choices that prioritize interoperability with commercial platforms, for example, can marginalize minority languages or local epistemologies. Procurement processes that privilege large-scale vendors often limit community governance or educator autonomy. In this systemic sense, digital ecosystems function not as instructional scaffolds but as developmental coordinators—they determine which pathways of participation become feasible and which forms of knowledge are sidelined. Their influence precedes individual learning encounters; it shapes the horizons within which teachers, students, and communities can act.

Alternative models demonstrate that infrastructures can be built around different principles. Estonia's X-Road framework retains jurisdictional control by securing data flows through distributed, encrypted in-country processing (Calzada, 2021). Uruguay's Plan Ceibal positions students and teachers as evaluators of educational technologies, embedding co-governance and public accountability into national design processes (Flecha, 2015). The Māori Data Sovereignty Network, Te Mana Raraunga, advances an approach rooted in kinship structures and collective stewardship, foregrounding relational accountability in digital governance (Table 1).

**Table 1:** Comparative models of digital governance and their underlying developmental principles.

Model	Principle	Mechanism
Estonia (X-Road)	National sovereignty	Encrypted, in-country data processing
Uruguay (Plan Ceibal)	Youth + teacher agency	Open-source platforms; participatory evaluation
Māori (Te Mana Raraunga)	Relational accountability	Collective governance rooted in kinship
Kenya (NEMIS/Huduma)	Surveillance-for-access	Biometric verification for education/services
Mexico (PPPs in ed-tech)	Digital dependence	Proprietary platform contracts tied to basic access

These differences reflect underlying values rather than mere technical contrasts. They answer foundational developmental questions: Who controls the architecture within which learning occurs? Who determines the legitimacy of knowledge? Whose futures are made actionable in code?

As scholars of platform governance argue, resisting platform domination requires institutional transformation rather than individualized digital literacy (Zuboff, 2019). Governance must therefore move upstream—toward infrastructural co-design, transparent procurement, and public stewardship. Uruguay's youth-run committees and participatory decision processes exemplify this shift from students as data sources to students as epistemic actors with influence over technological direction (Flecha, 2015). Extending the concept of the zone of proximal resistance, infrastructural participation enables youth and communities to reshape not only the artifacts of learning but the architectures that organize developmental possibility.

From this perspective, development is not delivered by digital systems but negotiated within them. Educational ecosystems must be intentionally designed to uphold cultural sovereignty, support dissent and experimentation, and maintain the multiplicity of epistemologies upon which diverse developmental trajectories depend. Without such commitments, digital infrastructures risk normalizing a future in which access is conditional, legibility is compulsory, and growth is constrained by what can be captured and classified.

*If digital ecosystems shape the material conditions of developmental life, translating these commitments into practice requires governance frameworks capable of supporting epistemic plurality and relational accountability*.

### 3.2 Policy and governance frameworks

*Effective governance of developmental surveillance demands more than reactive regulation; it requires structural commitments to what might be described as intergenerational sovereignty—the capacity of communities to shape the developmental conditions inherited by future cohorts—alongside epistemic justice and cultural legitimacy*.

Traditional data-protection frameworks—such as the General Data Protection Regulation (GDPR)—provide important baselines for transparency and data minimization. Yet they often rely on liberal-individualist assumptions that insufficiently capture collective, relational, or youth-centered developmental realities (Cannataci, 2021; Steeves and Regan, 2014). Because youth development is socially mediated and culturally situated, governance must address developmental dignity: the right of young people to grow in environments that support experimentation, autonomy, and emergent subjectivity rather than confining them within predictive templates.

In response to these limitations, governance models are increasingly shifting toward anticipatory and pluralist orientations. Such approaches emphasize collective stewardship, deliberative accountability, and context-sensitive regulation rather than solely individual consent-based mechanisms. These frameworks reimagine privacy not as a matter of isolated rights, but as a shared ethical responsibility—particularly in contexts marked by extractive histories and epistemic dispossession (Philip et al., 2011). Institutionally, this entails embedding participatory processes and culturally responsive evaluation mechanisms into the design and oversight of digital systems.

Embedding youth participation within policy infrastructures is equally crucial. Mechanisms such as participatory evaluation processes, open governance platforms, and youth advisory bodies enable young people to contribute to decision-making regarding educational technologies and data practices (as seen in participatory educational technology initiatives). Here, youth are engaged not simply as data producers but as contributors to the deliberative processes guiding technological design. Such arrangements shift governance from a model of external regulation to one of co-authored oversight.

Hybrid governance structures also offer promising pathways. Institutional designs that integrate community-based authority with formal regulatory systems—such as co-governance arrangements and culturally grounded data stewardship frameworks (e.g., Māori data sovereignty models)—enable governance to remain responsive to local epistemologies while maintaining broader accountability. By institutionalizing collective stewardship, such systems preserve the Vygotskian insight that development emerges through culturally embedded relationships rather than abstract computational assessment.

Yet enforcement environments differ significantly across regions. Where governance frameworks include independent oversight bodies, enforceable standards, and public accountability mechanisms, they are more likely to preserve conditions for relational trust and developmental autonomy. Conversely, weak or fragmented regulatory environments allow digital platforms to shape learning conditions without sufficient scrutiny. These divergences underscore that governance is itself a developmental condition: the institutional arrangements surrounding digital systems directly influence how young people experience learning, agency, and identity formation.

For educators, these shifts require more than technical competence. Teachers increasingly find themselves mediating between platform metrics and student well being—interpreting algorithmic outputs, adjusting data, or quietly resisting reductive scoring. Professional development must therefore include critical data literacy, enabling educators to recognize when systems constrain rather than support learning. Classroom practice can reclaim interpretive authority by prioritizing dialogic assessment, student self-evaluation, and collective deliberation over automated scoring. In this sense, pedagogical agency becomes a form of governance: the capacity to shape the developmental conditions that technology alone cannot determine.

Institutionalizing effective governance therefore requires more than legislation—it requires inclusive infrastructures. Core mechanisms include public audit systems, independent oversight authorities, youth ombudsperson roles, and avenues for collective legal redress. These tools enable ongoing scrutiny of technological systems and provide pathways for contestation and revision. In this sense, governance becomes an iterative process rather than a fixed regulatory framework, capable of adapting to evolving technological and social conditions.

Youth are not only responding to surveillance—they are increasingly shaping the norms that govern it. Through advocacy, participatory forums, and contributions to international policy discussions, young people are emerging as active agents in defining ethical standards for digital systems. These interventions mark a shift from reactive resistance toward proactive normative authorship, extending the concept of developmental agency into the domain of governance itself.

A diverse toolkit of emerging and proposed policy instruments is taking shape to support this transition. These include binding privacy-by-design standards embedded in procurement processes (Cavoukian, 2009), enforceable algorithmic audit rights, cultural impact assessments that evaluate alignment with local values, and formal mechanisms for youth representation in decision-making bodies.

Fiscal instruments—such as allocating portions of platform-generated revenue toward public digital infrastructure—can further support equitable access and accountability, functioning as redistributive mechanisms within digital governance systems. Together, these instruments translate normative commitments into operational governance structures.

Taken together, these mechanisms form what can be understood as a relational governance ecology—an integrated framework that aligns institutional design with youth autonomy, communal accountability, and cultural sovereignty. Such an approach repositions governance not as an external constraint but as a constitutive dimension of developmental environments.

Ultimately, governance must be understood as a dynamic and participatory system, shaped across generations. Institutional innovations such as youth–elder councils, participatory budgeting mechanisms, and layered jurisdictional models illustrate how authority can be distributed and negotiated rather than imposed. If contemporary surveillance practices function as a curriculum of power, then governance must operate as a pedagogy of accountability, recognition, and intergenerational authorship—re-establishing the conditions under which development can proceed with dignity, plurality, and cultural depth.

*Yet infrastructures and regulations alone cannot explain the full trajectory of developmental surveillance. Their meanings and consequences are also shaped by cultural interpretation, collective resistance, and emerging technological futures*.

## 4 Transformative horizons: culture, resistance, and futures

### 4.1 Cultural perspectives on developmental surveillance

*Surveillance systems are never reducible to their technical design or legal regulation; they are also interpreted through culturally specific contexts that shape how they are implemented, contested, and made meaningful*.

These technologies are never culturally neutral; they carry with them inherited logics of authority, hierarchy, and political ideology that shape their reception across contexts. What Philip et al. (2011)et al. ) describe as digital recolonization persists as platform infrastructures apply dominant epistemologies—classifying, sorting, and normalizing youth according to globally standardized categories that obscure local knowledge and relational practices. Such lenses not only influence adoption but define what is publicly recognized as development, responsibility, or maturity.

In settler-colonial contexts such as Australia, biometric monitoring in Indigenous communities raises concerns about the reactivation of assimilationist schooling practices, where cultural divergence is framed as risk rather than richness. In East Asia, disciplinary technologies resonate with long-standing civic traditions: China's keju legacy and broader Confucian ideals are sometimes invoked to frame the cultural legitimacy of emotion-sensing AI and posture-monitoring classrooms. These infrastructures moralize discipline as virtue, shaping developmental expectations through deeply embedded ethical frameworks. From the perspective of youth formation, they recalibrate what it means to inhabit social roles, embedding preferred conduct into the routines of daily life.

Resistance to these systems also emerges from culturally specific ontologies. For some Navajo community members, biometric identification has been interpreted as violating hózhó—an ethic of balance, beauty, and relational integrity. Practices such as morning corn pollen offerings—embedded in relational cosmologies—highlight tensions with disembodied forms of biometric abstraction. In Pakistan and Brazil, Islamic and Catholic traditions have been invoked in public debates to provide theological grounds for asserting bodily sanctity and critiquing extractive surveillance. In Ghana and related contexts, anti-colonial arguments challenge foreign ed-tech models that attempt to standardize learning through distant metrics (Philip et al., 2011). Such responses reveal that refusal is never merely technical; it is shaped by locally anchored visions of what constitutes a flourishing developmental trajectory.

Across many documented settings, digital systems have been introduced in contexts where material conditions remain precarious. Instead of repeating the familiar critique that predictive analytics “substitute” for development, it is more accurate to say that technological systems often arrive faster than the infrastructures needed to sustain their promises. Documented cases indicate that in settings such as rural Nigeria or Peru, students may complete AI-proctored exams in environments with intermittent electricity. Here, computational oversight becomes embedded in contexts that lack stable educational resources, transforming surveillance into a compensatory mechanism rather than a complementary one. This dynamic exposes a broader inequity: in regions where social investment is thin, algorithmic governance becomes thick.

Alternative models of technological governance grounded in African, Indigenous, and postcolonial epistemologies reveal different ways of aligning infrastructure with cultural meaning. Botswana's kgotla tradition emphasizes deliberation and collective judgment; Bolivia's Vivir Bien doctrine foregrounds ecological ethics and relational accountability; South Korea's norms of intergenerational trust shape consent and oversight. These examples do not exhaust internal debate within these societies but illustrate distinct approaches to aligning technological governance with culturally embedded values. These are not peripheral adaptations of Western digital-rights discourse but expressions of epistemic authorship—community-driven determinations of when and why surveillance is legitimate (Diagne, 2016; Sarr, 2019). All of these models underscore that development is mediated through culturally specific expectations about interdependence, responsibility, and adulthood.

As Souleymane Bachir Diagne (2016) reminds us, developmental trajectories are “plural horizons of dignity.” In Seoul, development may imply progress through optimization technologies; in Navajo contexts, it may signify ceremonial continuity; in rural Ghana, it may entail active refusal of intrusive systems. Such trajectories embody self-authored cultural aims. Yet refusals must not be romanticized: communities often resist not from idealized autonomy but from histories of marginalization, structural neglect, or the costs imposed by misaligned infrastructures.

UNESCO frameworks have supported Bolivia's use of community-based benchmarks, while Norway's educational policies recognize Sámi duodji as evidence of learning within public education. These examples enact relational governance—affirming that communities have the authority to define trust, evaluation, and growth on culturally resonant terms (Lopez and Singh, 2024). In developmental theory, this aligns with Vygotsky's emphasis on social mediation: learning emerges within shared cultural practices rather than through disembedded, automated assessment.

A brief historical reflection can illuminate what is culturally distinctive about contemporary algorithmic governance. Earlier developmental systems—most notably the Soviet youth organizations—have already been introduced in this manuscript, but here they serve a different purpose: to highlight how state-led developmental projects can mobilize cultural narratives and collective ritual rather than computational measurement. What is notable in the Soviet case is not efficiency or moral clarity, but the cultural grammar through which youth formation was pursued: narrative teleology, civic mythology, and communal mentorship. This comparison underscores that all developmental governance systems are culturally situated; what varies is the medium—ritual or algorithm, narrative or prediction—not the fact of cultural embedding itself.

This raises a broader question for the present: how might societies cultivate developmental ethics that are future-oriented without collapsing into technocratic determinism or ideological uniformity? Post-socialist and decolonial epistemologies point toward models grounded in relational accountability. Historical examples such as Cuba's literacy brigades pursued development through collective responsibility, emphasizing coordination and care rather than behavioral capture. Their significance lies not in nostalgia but in illustrating that alternative infrastructures of youth formation are possible, while remaining attentive to the political and historical complexities within which they emerged.

To build globally just algorithmic systems, we must attend not only to technical architectures but to the cultural visions of development they implicitly advance. The challenge is not to standardize youth through data, but to ensure that digital systems remain accountable to the communities they affect. Development, in this view, becomes a culturally grounded practice of authorship—a process shaped by memory, narrative, and collective aspiration, rather than a computational category to be measured or optimized.

*These same cultural frameworks also shape how young people contest surveillance through everyday practices of evasion, reinterpretation, and collective agency*.

### 4.2 Youth resistance and reimagined agency in the biometric age

In an age of pervasive surveillance, youth are not passive recipients of control but active creators of resistance. While biometric systems seek to optimize behavior through ambient modulation, young people respond with strategies that span technical evasion, social solidarity, and embodied subversion. Their actions transform constraint into critique, revealing what this paper terms zones of proximal resistance. Adapted from Vygotsky's (1978) notion of the zone of proximal development, this concept describes the relational space in which youth learn resistance practices through social mediation, shared literacies, and collective experimentation. Just as Vygotsky argued that cognitive growth emerges through guided participation, resistance too is co-constructed—taught, refined, and transmitted through peer networks and cultural communities.

Technical resistance ranges from spoofing biometric metrics to encrypted peer networks. In Brazil, favela teens have been reported to construct or experiment with Tor-enabled mesh relays from salvaged hardware to mask their digital presence during online schooling. In Ukraine, youth have repurposed gaming platforms and communication channels into coordination tools during military conflict, demonstrating how familiarity with digital ecosystems can be mobilized under crisis (Aviv and Ferri, 2024). Recent cases in Australia, where youth have bypassed age-verification systems through VPNs and identity workarounds, further illustrate the adaptability of resistance practices. These tactics exemplify forms of infrastructural creativity, often nurtured within ecosystems of trust, mutual aid, and informal apprenticeship.

Social and cultural strategies broaden these repertoires. Across diverse contexts, youth adopt linguistic and semiotic tactics—ranging from coded memes to hybrid language use—to evade content moderation and algorithmic scrutiny. In Nigeria, Ugoala (2020) shows how Nigerian English memes encode subversive meanings that often escape platform detection. Educational research similarly documents how students repurpose or modify school-issued devices to extend access among excluded peers, from using shared logins to installing covert messaging tools (Selwyn, 2021). These practices illustrate what might be understood as algorithmic double consciousness: a cultivated awareness of how machine perception constructs legibility, coupled with strategic modulation of one's digital visibility. Drawing on Du Bois (1903) notion of “second sight,” this dual perspective enables youth to navigate systems of surveillance without fully capitulating to their terms.

To clarify the range of these strategies, the paper offers a taxonomy of youth resistance tactics. This provisional taxonomy is a theoretically informed synthesis—derived from documented studies of youth resistance (e.g., Selwyn, 2021; Shade and Singh, 2016; Philip et al., 2011) and inductive coding of recurrent tactics in digital subcultures—rather than a strictly empirical classification. It is not exhaustive, but organizes observed and plausible practices within contemporary youth digital cultures (Table 2).

**Table 2:** Taxonomy of youth resistance tactics in digitally surveilled environments.

Tactic type	Description	Illustrative example
Digital Camouflage	Masking behavior through diversion	Social posts mimicking gait spoofing to mislead algorithms
Parasite Platforms	Covert uses of benign apps	In-game chats repurposed for private communication
Semantic Subversion	Hybrid idioms to disrupt NLP classification	English–Yoruba meme blends in Nigerian digital culture
Sensorial Jamming	Obscuring or interfering with biometric input	DIY thermal masking, patterned makeup, visual occlusion
Algorithmic Evasion	Behavioral adjustments to mislead detection	Modified routines to confuse emotion-sensing AI systems

Resistance is also embodied. In Canada, neurodivergent students report adapting their behaviors during AI-based remote proctoring—masking eye movements, minimizing physical tics, or maintaining rigid posture to avoid algorithmic misclassification (Hull et al., 2017). These adaptations mirror broader patterns of autistic camouflaging, where individuals consciously or unconsciously suppress neurodivergent traits to conform to normative expectations. Such practices reclaim the body as a site of interpretive agency, exposing the assumptions about “proper” behavior encoded in surveillance infrastructure. At the same time, critics document that tools like Proctorio disproportionately penalize disabled students, revealing structural bias within remote proctoring systems (AI Incident Database, 2021).

Yet resistance is not without cost. Youth describe “performance exhaustion” and identity fragmentation—a condition Shade and Singh (2016) associate with the psychological toll of constant self-monitoring in surveilled environments. For neurodivergent and racialized students especially, the pressure to self-regulate under algorithmic scrutiny can produce estrangement from one's affective rhythms. These embodied dislocations echo Lauren Berlant (2011) notion of slow refusal: not overt defiance, but a quiet reorientation of compliance toward care, introspection, and survival (West, 2023).

Crucially, resistance is unevenly distributed. In Kenya, fingerprint-based identification systems in schools and public services reinforce longstanding racialized and socioeconomic hierarchies, with youth in marginalized communities facing the greatest burdens and the narrowest opportunities for evasion (Weitzberg, 2020). Such asymmetries reflect broader inequities in infrastructure, digital literacy, and cultural legitimacy. As curriculum reforms in contexts such as Finland suggest, resistance literacy should be cultivated not merely as a technical skill set but as an ethical and civic capacity: the ability to reconfigure, reinterpret, or refuse imposed systems (Sahlberg, 2021).

Supportive infrastructures amplify these forms of agency. In Barcelona, youth participate in co-designing municipal privacy tools (Calzada, 2021), and in Uruguay, Plan Ceibal grants students veto rights over algorithmic tools in public education (Flecha, 2015). These initiatives shift resistance from individualized tactics to collective authorship, operationalizing Vygotsky's insight that agency is socially scaffolded—emerging from relationships of trust, experimentation, and shared deliberation.

Youth resistance thus reveals a paradox: the more totalizing a system becomes, the more it incubates its own alternatives. These acts do not simply defy surveillance—they reimagine development itself. The challenge for pedagogy is not to extinguish refusal but to consider how its possibility might be institutionally sustained, ensuring that opacity, creativity, and dissent remain central to the unfolding of youth identity and autonomy. These practices of resistance unfold within institutional environments that increasingly structure learning itself.

*These present-day struggles also anticipate future conflicts over agency and autonomy as neurotechnologies, predictive systems, and immersive environments deepen the reach of developmental governance*.

### 4.3 Futures, neurotechnologies, and predictive governance

Emerging neurotechnologies, genomic tools, and immersive systems increasingly shape the conditions under which youth learn, emote, and imagine themselves. These tools no longer operate merely as extensions of existing data practices; they recast the boundaries between monitoring, intervention, and developmental design. What once appeared as observation now takes the form of anticipatory adjustment—calibrating behavior and affect in real time and normalizing this modulation through narratives of personalization, optimization, and enhancement.

Affective neurotechnologies exemplify this shift. EEG headbands experimentally introduced or marketed for educational use to enhance focus or emotional stability claim to infer frustration or attentional lapses from neural activity, inviting educators or institutions to respond based on interior states that are only partially understood (Ruckenstein and Schüll, 2017). Despite their pedagogical framing, such tools risk collapsing cognitive diversity into psychometric signals and obscuring structural inequities—including poverty, racialization, or inaccessible learning environments—beneath the veneer of neurobiological explanation. They advance a form of determinism in which neural signals substitute for relational or contextual interpretation.

Genomic technologies introduce an additional axis of categorization. Developmental “risk scores,” derived from polygenic or epigenetic data, are being explored in research and commercial contexts as potential inputs into targeted interventions, eligibility assessments, or educational pathways—often without meaningful oversight. Commercial platforms now offer parent-facing genetic evaluations whose opaque methodologies echo earlier eugenic frameworks (Galton, 1883), updated through contemporary interfaces and the authority of data-driven inference (Eubanks, 2018; Zuboff, 2019). These developments blur the line between biological description and developmental prescription, shifting the locus of judgment from observable behavior to supposedly latent potential.

Immersive systems further complicate the developmental landscape. Emotion-responsive VR environments dynamically adjust visual or auditory stimuli to reinforce desired affective states such as calm, concentration, or compliance. Yet alternative deployments exist. Indigenous communities have begun developing augmented-reality platforms rooted in land-based pedagogy, oral tradition, and intergenerational storytelling. These contrasting uses reveal deeper epistemological tensions: whether immersive tools should support relation, memory, and ecological situatedness—or further standardize desirable emotional states (Haraway, 2016).

To navigate these emerging terrains, the paper articulates three interconnected dimensions of anticipatory governance. Drawing from developmental ethics, privacy theory, and emerging AI scholarship, these dimensions offer a framework for assessing how nascent technologies reshape the terms of becoming:

- Cognitive and emotional integrity—safeguarding the inner domain of thought, imagination, and affect from intrusive inference or manipulation, ensuring that internal states remain subject to relational interpretation rather than algorithmic adjudication.
- Relational and cultural continuity—ensuring that developmental environments remain grounded in community norms, cultural traditions, and intergenerational ethics, rather than being subordinated to external logics of optimization or risk management.
- Epistemic and infrastructural co-determination—enabling youth and communities to participate in shaping the data standards, design histories, and institutional rules that structure emerging systems.

These dimensions matter because they preserve what Vygotsky (1978) considered foundational: the cultural and relational contexts through which learning and identity evolve. Emerging technologies influence not only what young people can do, but the developmental worlds they inhabit.

Governance innovations already point toward possibilities for anticipatory regulation. Chile's proposals to constitutionalize neuro-rights aim to restrict the commodification of brain data and recognize mental privacy as a protected category (Cannataci, 2021). Participatory digital inclusion initiatives allow youth and communities to oversee or contest the introduction of new technologies in educational settings (Flecha, 2015). These efforts show how ethical principles can be embedded at the infrastructural level, rather than appended after harm occurs.

Culturally grounded governance will be essential as these technologies expand. Botswana's dikgotla councils, grounded in botho—a relational ethic of personhood—emphasize dialogue and respect over performance metrics (Diagne, 2016; Sarr, 2019). In such contexts, technologies that amplify storytelling, oral heritage, or collective reflection may carry greater legitimacy than systems that quantify affect or impose productivity norms. Barcelona's participatory procurement processes have been associated with forms of anticipatory refusal, including the rejection of certain affective monitoring tools in public-school contexts. These cases underscore that developmental futures are culturally authored: different societies prioritize different forms of care, knowledge, and relationality.

Technological foresight must therefore embrace the speculative not as technofuturism, but as a disciplined practice of imagining alternative developmental trajectories. The Māori Data Sovereignty Network, Te Mana Raraunga, articulates data governance rooted in collective agency and distributed authority, advancing models such as tribally governed, revocable consent frameworks. Algorithmic impact assessments should likewise expand beyond questions of bias to evaluate cultural distortion, relational disruption, and epistemic incongruence (Lopez and Singh, 2024). Such tools ground futures thinking in ethical, ecological, and cultural accountability.

Educational systems will need to prepare young people not only to navigate these emerging infrastructures but to shape and critique them (Selwyn et al., 2019). Curricula incorporating design justice, adversarial thinking, and epistemic pluralism can cultivate futures literacy: the ability to analyze, reimagine, and reconfigure the developmental conditions shaped by technology (Freire, 1970). This literacy is not merely technical—it is ethical and political.

Within this broader anticipatory framework, predictive analytics introduces a distinct but related set of challenges, centered on how developmental trajectories are inferred, stabilized, and acted upon over time. Predictive classification technologies increasingly claim authority over developmental potential. These tools do not merely register behavior—they construct trajectories through which youth are governed before actions unfold. Following Foucault (1977), this is biopolitical governance: the conversion of behavioral surplus into prediction products that reframe possibility as probability (Zuboff, 2019). When attentional metrics, linguistic patterns, or demographic proxies label students as “low aptitude,” the effect is not diagnostic but structuring—shaping expectations, opportunities, and institutional responses in advance.

Under these logics, the temporal and relational elasticity essential for developmental growth becomes compressed. Moments of hesitation, associative drift, or imaginative exploration—often catalysts for creativity—are reinterpreted as inefficiency or risk. Where developmental time might otherwise unfold through contingent encounters or interpretive dialogue, predictive infrastructures press toward linearity, smoothing uncertainty into manageability. This shift reconfigures developmental temporality by prioritizing probabilistic anticipation over open-ended social mediation.

Marginalized youth disproportionately bear the burden of these dynamics. Black linguistic innovation is misjudged as low literacy (Benjamin, 2019); Indigenous ecological knowledge is rendered invisible to standardized aptitude models; neurodivergent forms of communication or stimming are flagged as anomalies. These are not isolated misreadings but instances of epistemic subtraction: the filtering out of forms of life and meaning that do not conform to dominant interpretive grids. Predictive analytics thus produce not only biased outcomes but ontological compression, generating a reductive abstraction—a “data double”—that shadows the lived complexity of a child.

To mitigate these forms of compression, ethical design must work in tandem with anticipatory governance. Three interrelated orientations can help safeguard developmental plurality:

First, the protection of interpretive space. Rather than relying solely on opacity, this orientation emphasizes the preservation of interpretive multiplicity—the maintenance of environments in which behaviors can be understood relationally rather than reduced to algorithmic outputs. This requires design choices that limit reductive behavioral scoring and prioritize contextual explanation.

Second, temporal due process. Predictive assessments involving minors should remain provisional, revisable, and open to contestation. Institutional mechanisms—such as mandatory justification reports, audit trails, and review triggers—are necessary to ensure that predictions do not solidify into developmental constraints. Temporal due process preserves the contingency essential to growth (Hoofnagle et al., 2019).

Third, pedagogies of interpretive resilience. As a complement to futures literacy, educational environments should cultivate the capacity to interrogate predictive classification itself. Drawing on Freire's (1970) conscientização, such pedagogies enable learners to analyze how models frame their behaviors, recognize prediction as socially constructed, and assert agency in contesting algorithmic narratives.

Together, these orientations converge in a broader ethical stance that resists the pull of predictive certainty. Rather than seeking to perfect prediction, they foreground the importance of temporal elasticity, relational interpretation, and cultural depth in sustaining developmental life.

Unchecked, predictive systems risk moralizing divergence—treating creative disruption, reflective pauses, or unconventional learning rhythms as deviations to be corrected. Yet developmental research consistently shows that growth is often catalyzed by such irregularities. Protecting these moments is not indulgence; it is a developmental and democratic necessity.

Ultimately, the task is not merely to regulate emerging technologies but to secure a future in which youth retain the capacity to author the terms of their becoming. A just developmental horizon is one in which infrastructures support—not overwrite—the plurality, contingency, and imaginative reach that make human development irreducibly open-ended.

*Across culture, resistance, and technological futures, a common question emerges: whether youth development will remain an open-ended human process or become increasingly governed through prediction, optimization, and machine legibility*.

## 5 Conclusion

This paper has argued that digital surveillance has become a pervasive system of developmental governance, reshaping the very conditions under which young people grow. These transformations must be understood specifically as effects of surveillance infrastructures, rather than digital systems in general. The landscape of youth development is being reshaped by systems that reach deeply into everyday life—not as peripheral tools but as structuring forces that participate in how learning, identity, and intergenerational relations unfold. Across the domains examined, developmental surveillance reorganizes the relational, cultural, and institutional environments within which young people orient themselves.

These influences do not operate uniformly. Some systems translate social interaction into behavioral scores; others infer emotional states or anticipate future actions; still others reshape the institutional architectures that regulate access to resources, participation, and legitimacy. Taken together, these processes alter the conditions under which development becomes possible—shifting how youth interpret their possibilities, navigate institutional demands, and craft meanings from experience. Yet, as the analysis has shown, these transformations are neither total nor uncontested. Young people engage in strategies of adaptation, refusal, creativity, and reinterpretation, demonstrating that development remains a negotiated and co-authored process rather than a fully programmable trajectory.

The central contribution of this paper lies in conceptualizing surveillance not as a set of discrete technologies but as a form of developmental governance. From this perspective, the question is not only how systems monitor behavior, but how they structure the environments in which learning, identity formation, and social participation unfold. Developmental governance foregrounds the ways in which surveillance infrastructures organize possibility itself—shaping what can be recognized, valued, and pursued within institutional and cultural contexts.

The challenge, then, is not simply to mitigate harm, but to cultivate governance arrangements capable of sustaining developmental plurality within surveilled environments. This requires moving beyond generic regulatory templates toward frameworks responsive to cultural specificity, collective ethics, and intergenerational responsibility. Safeguards such as contestation rights, limits on high-stakes prediction, and protections for mental privacy remain essential, but they must be complemented by deeper institutional commitments: community authority over technological design, locally grounded definitions of care and responsibility, and participatory oversight that includes youth as active contributors.

For educators, these dynamics carry practical implications. Teachers increasingly mediate between platform metrics and student well being, interpreting algorithmic outputs while preserving relational and dialogic forms of learning. Professional development must therefore include critical data literacy alongside pedagogical responsiveness. Classroom practice can reclaim interpretive authority by prioritizing dialogic assessment, student co-evaluation, and collective deliberation over automated scoring.

Decentralizing design authority is central to this work. When commercial or state systems dictate how youth are represented or evaluated, the result is a constrained vision of development. By contrast, when communities, educators, and young people themselves participate in shaping the infrastructures they inhabit, developmental environments can sustain exploration, relational depth, and cultural continuity. In this sense, education can function, in Freire's (1970) terms, as a practice of collective reflection and world-making rather than an arena of compliance.

At the same time, developmental governance must remain attentive to the material and ecological conditions that sustain digital infrastructures. The extraction of resources, the distribution of computational power, and the geopolitics of data storage all shape the environments in which youth live and learn. A critical account of surveillance must therefore situate developmental processes within these broader material and ecological relations, without losing sight of their grounding in everyday social experience.

What, finally, do we owe the next generation in an age of pervasive surveillance? Not a curated developmental pathway, but the conditions under which they can participate in shaping their own trajectories. Not predictive certainty, but the preservation of interpretive space, relational meaning, and cultural variability. As Vygotsky's framework reminds us, development is always co-authored—emerging through social mediation, shared practices, and evolving cultural contexts. To sustain that co-authorship is to recognize young people not as objects of optimization, but as agents of meaning, inheritors of histories, and participants in futures that cannot be fully anticipated or computationally prescribed.

The task is not to optimize youth development, but to preserve its openness—its capacity to remain relational, interpretive, and irreducibly unfinished.
